# Henri Coutard (1876–1950): A Pioneer of Fractionated Radiotherapy Regimens

**DOI:** 10.7759/cureus.69371

**Published:** 2024-09-13

**Authors:** Aaditya Prakash, Amitabh Kumar Upadhyay, Abhishek Kumar

**Affiliations:** 1 Radiation Oncology, Tata Main Hospital, Jamshedpur, IND; 2 Medical Oncology, Tata Main Hospital, Jamshedpur, IND; 3 Nuclear Medicine, Tata Main Hospital, Jamshedpur, IND

**Keywords:** external beam radiation therapy, fractionated radiotherapy, henri coutard, laryngeal cancer, protracted-fractionated radiotherapy

## Abstract

Henri Coutard was a prominent French roentgen therapist (radiation therapist). He was a pioneer physician and scientist who established the development of the “protracted-fractional method” of radiation dosing. He treated laryngeal cancer with super-voltage x-rays and also published data on five-year survival rates while his contemporary physicians indulged in treating cancer with radioactive sources. Due to his significant contribution regarding the use of fractionated dose regimens to treat cancer, in today's world most of the tumors are treated using the same principle which is in itself a great achievement in the field of radiation oncology. Due to his work, present-day radiotherapy practice is focused on therapeutic ratios to treat cancer.

## Introduction and background

Henri Coutard's incredible contribution to the discovery of fractionated radiation therapy in the treatment of laryngeal cancer is widely recognized. Following his military service in World War I as head of radiologic services at the military evacuation hospital in Baccarat (France), he joined the Radium Institute of Paris. Here he did his research work with experimental irradiation to lower animals. He also excelled in roentgen therapy as well as radio diagnostic procedures. He worked on the treatment of inoperable upper airway cancer with supervoltage x-rays. He and Regaud and Nogieur noted what they termed “Radioepidermitis,” which is radiation-induced epidermis exfoliation and dermal denudation. Similarly, “Radioepithelitis” was used to describe the radiation-induced reaction in the oro-mucosal membrane. He along with his colleagues also treated cases of “Lymphoepithelioma” and other carcinomas. He also observed that the radiosensitivity varies among tumors, so they respond differently even in the same dose of radiation.

In 1930, he relocated to Chicago Tumor Institute (USA) and treated Mr. Penrose, a well-known entrepreneur who had been diagnosed with esophageal cancer as a secondary malignancy. He initially treated Mr. Penrose for laryngopharyngeal cancer in Paris in 1930.

Coutard's final experiments were deemed unconventional by his colleagues, but his earlier work helped to establish radiation therapy as a recognized cancer treatment. According to Grigg, Coutard's most significant contribution was “teaching a generation of radiologists to carefully observe their patients and painstakingly record the clinical course of treatment.” Apart from his 1949 monograph, he authored approximately 35 papers during his life.

## Review

Early life

On August 27, 1876, Henri Coutard was born in the historic French town of Marolles-les-Braults, which is located 25 miles north of Sarthe. His mother, Melanie Marie Josephine Coutard, was a merchant of novelty, and his father, Louis Coutard, was a local government employee in charge of the agricultural community. He had a younger sister named Helene and an older brother named Louis [1,2].

Education, career in military service, and marriage

Coutard enrolled as a boarding student at the Lycee Montesquieu of Le Mans in 1887, at the age of eleven. He studied there for the next seven years. He was awarded two baccalaures: one in letters in 1893 and one in mathematics in 1894. For his achievements in the gymnastics and military programs at his school, he was also given an award by the war minister.

His medical education began at the University of Paris after he finished secondary school, where he received training in Parisian hospitals and finished an internship in Nantes. “Extraperitoneal lesions of the bladder and rectum observed in cases of pelvic fracture” was the title of his doctoral thesis. In addition to one from his prior patients, it summarized eight cases from the literature. He presented his thesis to the Faculty of Medicine tribunal on July 17, 1902 [1]. Following graduation from medical school, Coutard joined the elite French Army Mountain infantry force, the Chasseurs Alpins [1]. He moved to the Jura Mountains to recuperate from pulmonary tuberculosis, practicing general medicine and curing his mild case of the disease during this time [2].

Coutard was the head of radiologic services in a military hospital near Baccarat, Meurthe-et-Moselle, on the Eastern Front, following his enlistment in the military during World War I [1,3,4]. Coutard met Claudius Regaud, a well-known radiobiologist, he would later collaborate with, there. Both developed the concept of “fractionation” and demonstrated that dividing the larger or total dose of radiation into much smaller fraction treatment schedules over weeks could help in achieving the curative dose to the tumor causing less toxicity to normal tissue.

Additionally, Coutard served in a radiological ambulance unit under Marie Curie [1,5]. At the close of World War I, on March 25, 1919, Coutard married Anne-Marie Adèle Rougier in Paris at the age of 43 [1,2].

Radium Institute, Paris, France 

After becoming interested in the possible medical applications of radioactivity, Coutard left for Paris in 1912 [1-3]. At an experimental laboratory co-founded by physicist Jacques Danne, Coutard started researching the properties of radium [1,2]. In 1912, he gave a presentation on the findings of his research at the French Association for the Advancement of Science meeting. His work concentrated on the therapeutic uses of radon gas in animals.

As the head of the x-ray division at the University of Paris' Radium Institute in 1919, Coutard collaborated with scientists such as Regaud and Antoine Lacassagne [2,5]. He performed radiation therapy on patients, carried out animal experiments, and carried out diagnostic imaging of the larynx and pharynx using a single -ray machine in the institute's basement [5,6]. In his early research during this time, he noticed that tumors that were not sufficiently irradiated would recur. He also observed that after a few weeks of radiation therapy, the Oro-pharyngeal mucous membrane gets inflamed and appears like diphtheroid pseudo-membranes, which he termed “radioepithelitis.” For this reaction, the radiation dose must have been sufficiently high to cause the mucous membrane to appear to react and to get inflamed [1]. Reports from six laryngeal cancer patients treated by Coutard with radiation therapy were presented at the 1921 International Congress of Oto-Rhino-Laryngology in Paris. Following the public recognition of his work, radiation therapy became the cornerstone of cancer treatment [2].

The optimal time to give radiation doses was a topic of debate among scientists [1]. In that era, some contemporary physicians were treating cancer with radioactive sources like radium. Coutard used x-ray in a range of several kilovoltages as supervoltage x-ray generators to treat cancer [7]. Coutard hypothesized that long radiation exposures spaced out over multiple weeks would yield the best effects because they would allow tissue to heal in between treatments [1,2]. At the 1928 International Congress of Radiology, he demonstrated his method, which became known as “Coutard's method” or the “protracted-fractional method” [1,6]. With this method, Coutard was able to treat laryngeal cancer for the first time with radiation therapy. He obtained data by 1930 regarding the five-year survival rate of laryngeal cancer patients. Treated with the help of the protracted-fractional method [6]. Using a radiometer, he built himself, he carefully documented the treatments he gave to each patient, even though he never published strict guidelines for radiation dosages [2,6]. During the course of the following ten years, he kept experimenting with various treatment plans, such as interrupted regimens and short, intense doses [6,8]. At the Radium Institute, Coutard trained and met with foreign radiotherapists, including Simeon T. Cantril, who later became the first president of the American Society for Radiation Oncology [9].

US experience

Coutard developed an interest in US radiation research after the development of supervoltage -ray (more than hundreds of kilovolts) generators, which could produce several hundred-kilovolt units [1]. Coutard received an invitation to work at the California Institute of Technology's Kellogg Research Laboratory from physicist Charles Christian Lauritsen, who was a mentor to Robert Andrews Millikan. Simultaneously, Coutard received an offer to take a leading role at the Chicago Tumor Institute from Max Cutler. Late in 1937, Coutard agreed to both offers [1]. François Baclesse took over at the Radium Institute after he resigned [6]. He collaborated closely with physicists Seeley G. Mudd and Millikan at Caltech, where he studied high-voltage therapy [1,2].

Following a six-month tenure at Caltech, Coutard relocated to Chicago, where he taught graduate courses at the Chicago Tumor Institute and conducted research on the application of brief, concentrated radiation dosages for the treatment of laryngeal cancer [2]. “The Great Depression” (1929-1939) tempered Cutler's aspirations for the institute [1]. The institute saw few patients, and he was unable to obtain a supervoltage unit for Coutard [1]. Around 1938, Coutard treated esophageal cancer in the American philanthropist and entrepreneur “Spencer Penrose,” whom he earlier treated for cancer of the laryngopharynx in Paris in 1932. In order to continue his treatment in the US, Penrose brought Coutard to Colorado Springs, Colorado, where he purchased a radiotherapy unit for his treatment [1]. In spite of all efforts, Spencer succumbed to cancer progression in late 1939.

In his will, Penrose stipulated that his radiotherapy equipment be donated to the neighboring Glockner Hospital, which is now Penrose Hospital, after his death in 1939. His wife Julie Penrose extended an invitation to Coutard to work at the recently opened Penrose Tumor Clinic at the hospital, which she paid for with contributions from their El Pomar Foundation [1]. He agreed, and in 1941 he relocated to Colorado Springs [2].

Career downfall

Coutard’s research became more random in the last 10 years of his life. He ceased publishing research in scientific journals and started carrying out unconventional experiments, such as using gold blocks as x-ray filters. He also proposed hypothetical theories about beta particles [2]. He grew more and more alone as a result of his peers criticizing his ideas [1]. In 1949, Coutard's monograph condensing his research from Colorado Springs was published [1]. Mostly disregarded by peers and respectable journals, historian and radiologist E. R. N. Grigg called the monograph a “rambling mixture of clinical observations, working hypotheses, and fantastic assumptions” [2].

Death and legacy

In late 1949, while visiting Copenhagen's Radium Station, Coutard ran into the director Jens Nielsen, one of his last surviving supporters [1]. He experienced an intracerebral hemorrhage in December 1949 while visiting his sister's family in France. On March 16, 1950, after a protracted illness, Coutard passed away at his sister's residence in Le Mans [1].

Because of Coutard's earlier contributions, radiation therapy for cancer patients is now widely accepted, even though his contemporaries disapproved of the experiments carried out in his final years. According to Grigg, Coutard's greatest contribution was “teaching a generation of radiologists to observe their patients carefully and to record painstakingly the clinical course of treatment” [2].

## Conclusions

Modern dose fractionation techniques have their roots in Coutard's prolonged-fractional radiotherapy regimen for cancer treatment. Coutard was among the first to recognize that different cancer histologies and locations carried distinct probabilities for radio curability. Coutard was renowned for being the pioneer in presenting research on x-ray imaging for both therapeutic and diagnostic purposes. Physicians from around the world trained at the Radium Institute during his period, and by the 1930s, interest began shifting away from radium therapy and toward x-ray-based radiation therapy. He was one of the first to present survival data for malignancies lasting more than 5 years. Because of his pioneering work, today's radiotherapy practice focuses on therapeutic ratios to treat cancer. His technological advances included many concepts taken for granted today, including custom immobilization of patients, beam hardening with metallic filters to achieve higher photon energies and collimation of beams. His contributions to the field of radiation oncology are the founding stones of the present-day multi-fraction radiation regimen. In his final years, he also experienced some setbacks. Still, he was not only a great patriot but also a competent physician.
